# Structural Dependence of Sulfated Polysaccharide for Diabetes Management: Fucoidan From *Undaria pinnatifida* Inhibiting α-Glucosidase More Strongly Than α-Amylase and Amyloglucosidase

**DOI:** 10.3389/fphar.2020.00831

**Published:** 2020-06-05

**Authors:** Hui Si Audrey Koh, Jun Lu, Weibiao Zhou

**Affiliations:** ^1^NUS Graduate School for Integrative Science and Engineering, National University of Singapore, Singapore, Singapore; ^2^Department of Food Science and Technology, National University of Singapore, Singapore, Singapore; ^3^Faculty of Health and Environmental Sciences, School of Science, Auckland University of Technology, Auckland, New Zealand; ^4^Faculty of Health and Environmental Sciences, School of Public Health and Interdisciplinary Studies, Auckland University of Technology, Auckland, New Zealand; ^5^Maurice Wilkins Centre for Molecular Discovery, Auckland, New Zealand; ^6^College of Food Engineering and Nutrition Sciences, Shaanxi Normal University, Xi’an, China; ^7^National University of Singapore (Suzhou) Research Institute, Suzhou, China

**Keywords:** fucoidan, *Undaria pinnatifida*, Diabetes, α-amylase, α-glucosidase, amyloglucosidase

## Abstract

Fucoidan refers to a group of sulfated polysaccharide that is commonly obtained from various species of brown seaweed. Fucoidan has gained increased popularity among researchers in the recent years due to its numerous biological activities, including its inhibitory effects against starch hydrolyzing enzymes such as α-amylase and α-glucosidase. This highlights the potential of fucoidan as an antidiabetic agent in the management and prevention of diabetes mellitus. In this study, the inhibitory effects of fucoidan isolated from the New Zealand *Undaria pinnatifida* seaweed species against three starch hydrolyzing enzymes—α-amylase, α-glucosidase, and amyloglucosidase—was investigated. It was demonstrated that while the fucoidan exhibited significant inhibitory effects against all the three starch hydrolases, it is an uncompetitive inhibitor of α-amylase and amyloglucosidase, and is a competitive inhibitor of α-glucosidase. Moreover, it exhibited significantly stronger inhibitory effects against α-glucosidase than α-amylase, thus having the desirable characteristics as an antidiabetic agent.

## Introduction

Diabetes mellitus is a chronic disease characterized by a high fasting plasma glucose concentration of ≥126 mg/dl (World Health Organization, 2003). There are two types of diabetes mellitus—Type 1 diabetes mellitus (T1DM) and Type 2 diabetes mellitus (T2DM)—of which approximately 90–95% of diagnosed diabetes cases are T2DM (American Diabetes Association, 2010). Patients with T2DM are often characterized by hyperglycemia, usually with a contribution of insulin resistance. In Singapore in 2010, 11.3% of the population aged 18 to 69 years old was diagnosed with diabetes, and diabetes was the 10^th^ leading cause of death (Ministry of Health, 2016). Moreover, due to the many long-term complications of diabetes including lower extremities amputations, cardiovascular disease (ischemic heart disease and stroke), and diabetic ketoacidosis, diabetes mellitus is a major health concern worldwide (Centers for Disease Control and Prevention, 2017).

Fucoidan refers to a group of sulfated polysaccharide isolated mainly from brown seaweed species, with α(1 3) and α(1 4) linked α-L-fucopyranose backbone (Li et al., 2008; Jiao et al., 2011). In the recent years, fucoidan has gained increased popularity among researchers due to its numerous bioactivities including its anticancer and antioxidant activity (Zhao et al., 2018). Fucoidan isolated from many different seaweed species has been reported to exhibit both primary and secondary antioxidant activity (Silva et al., 2005; de Souza et al., 2007; Lim et al., 2014). Likewise, fucoidan from *Undaria pinnatifida* has been demonstrated in the literature to exhibit inhibitory effects against a number of cancer cell lines including the A-549 lung carcinoma cell line, MCF-7 breast adenocarcinoma cell line, SK-MEL-29 melanoma cancer cell line, T-47D breast cancer cell line, and the WiDr colon adenocarinoma cell line (Vishchuk et al., 2011; Mak et al., 2014; Lu et al., 2018). However, it has also been reported in the literature that the bioactivities of fucoidan are dependent on numerous factors including the species of seaweed from which fucoidan is isolated from, the geographical location where the seaweed is harvested, the method of extraction, maturity and harvest period of seaweed, as well as the structure and chemical composition of fucoidan (e.g. sulfate content, monosaccharide content, and uronic acid content) (Li et al., 2008; Koh et al., 2019). As such, fucoidan isolated from different species of seaweed and from different geographical location is likely to possess different bioactivity (Li et al., 2008).

Beyond the antioxidant and anticancer activity, fucoidan from various seaweed species has been shown to exhibit different inhibitory effects against starch hydrolyzing enzymes such as α-amylase, α-glucosidase, and amyloglucosidase (Cho et al., 2011; Kim et al., 2014; Lakshmanasenthil et al., 2014; Kumar et al., 2015). Kim et al. (2014) reported that fucoidan isolated from *Ascophyllum nodosum* exhibited inhibitory activity against α-amylase and α-glucosidase, while fucoidan isolated from *Fucus vesiculosus* exhibited inhibitory activity against α-amylase only. Moreover, it has been reported that the starch hydrolase inhibitory activity of fucoidan is also influenced by its sulfate content. Cho et al. (2011) extracted fucoidan from the sporophyll of *U. pinnatifida* and chemically modified them to increase the level of sulfate content. They reported that over-sulfation of fucoidan enhanced the inhibition effect on amyloglucosidase but not on α-amylase (Cho et al., 2011). These results suggest that the inhibition effect of fucoidan depends on both its chemical composition as well as the type of starch hydrolase enzyme involved.

However, there are very limited research efforts on the type of inhibition and inhibition activity of fucoidan from the New Zealand *U. pinnatifida* seaweed on the various starch hydrolyzing enzymes. Fucoidan from *U. pinnatifida* possesses a unique backbone structure of alternating α(1–3) and α(1–4) galactose and fucose units, which has been associated with its superior anticancer activity against T-47D breast cancer cell line and SK-MEL-28 melanoma cancer cell lines than fucoidan from other seaweed species (Vishchuk et al., 2011; Lu et al., 2018). It is hypothesized that this fucoidan with its unique backbone structure and its monosaccharide composition of galactose and fucose at a ratio of approximately 1:1 may possess different inhibition activity and potency against the various starch hydrolyzing enzymes (Koh et al., 2019).

In addition, commercially available antidiabetic drugs such as acarbose are known to cause adverse side effects including abdominal distension, flatulence, meteorism, and diarrhea as a result of the strong inhibitory effects of acarbose against α-amylase (Puls and Keup, 1975; Bishoff, 1985). As such, existing research efforts are focused on characterizing natural extracts with strong α-glucosidase inhibition activity for hyperglycemia management and prevention.

In this study, fucoidan extracted from the New Zealand *U. pinnatifida* was investigated for its inhibitory activity against the three starch hydrolyzing enzymes i.e. α-amylase, α-glucosidase, and amyloglucosidase. The type of inhibition against the three different starch hydrolases was also determined *via* enzyme activity kinetic analysis using the Michaelis-Menten and Lineweaver-Burk models.

## Materials and Methods

### Chemicals and Fucoidan Samples

Fucoidan isolated from the brown seaweed species *U. pinnatifida* was obtained from Auckland, New Zealand (Bi et al., 2018). Corn starch, maltose, porcine pancreatic α-amylase, sodium phosphate buffer, calcium chloride, sodium potassium tartrate tetrahydrate, sodium hydroxide, 3,5-dinitrosalicylic acid, p-nitrophenol, 4-nitrophenyl α-D- glucopyranoside, and sodium carbonate were purchased from Sigma-Aldrich (Sigma-Aldrich, St. Louis, MO, USA). Amyloglucosidase and α-glucosidase were purchase from Megazyme (Megazyme, Bray, Ireland).

### Inhibition Assay for α-Amylase Activity

The inhibition activity of fucoidan against α-amylase was determined by measuring the reducing power of released maltose from soluble starch according to the method of Sui et al. (2016) with minor modifications. A series of tests on various substrate (corn starch) and inhibitor (fucoidan) concentrations were conducted to determine the inhibition type. The working enzyme solution was prepared freshly prior to use by solubilizing 1 mg of porcine pancreatic α-amylase in sodium phosphate buffer (SPB, 0.1 M, pH 6.9). The substrate solutions of various concentrations (2.5, 5, 10, 15 mg/ml) were prepared by solubilizing cornstarch into SPB and gelatinized at 100°C for 15 min. The substrate solutions were allowed to cool to room temperature prior to use. The fucoidan solutions of various concentrations (0.5, 1, 1.5, 2 mg/ml) were prepared by solubilizing the fucoidan samples in SPB. Calcium is an essential co-factor for enzyme α-amylase (Morris et al., 2011). Hence, the SPB used in these experiments were prepared freshly prior to use with 40 mg/L of calcium chloride added.

To prepare the color reagent solution, a 5.3 M sodium potassium tartrate solution was prepared by solubilizing sodium potassium tartrate tetrahydrate in a 2 M sodium hydroxide (NaOH) solution with constant stirring. The sodium potassium tartrate solution was heated and maintained at 50–70°C, and care was taken to avoid boiling of the solution. A 96 mM 3,5-dinitrosalicylic acid (DNS) solution was prepared by solubilizing DNS in deionized water. The DNS solution was heated and maintained at 50–70°C. Deionized water was heated to 60°C and the deionized water, sodium potassium tartrate solution, and DNS solution were allowed to mix slowly in the ratio of 3:2:5. The resulting mixture was stirred to ensure complete dissolution. The working color reagent was stored in an amber flask at room temperature for use.

Aliquots of fucoidan solution of 20 μl and α-amylase solution of 20 μl were added into a 2 ml Eppendorf tube and incubated at 37°C water bath for 15 min to allow for interactions between the enzyme and fucoidan. The enzyme reaction was initiated by adding 60 μl of starch solution into the Eppendorf tube containing the enzyme-fucoidan reaction mixture. The reaction was allowed to proceed for 5 min at 37°C. Subsequently, 100 μl of DNS color reagent was added into the Eppendorf tube and incubated at 100°C for 15 min to allow for color development. The reaction mixture was then cooled in an ice water bath. Aliquots of 200 μl of reaction mixture were transferred into a 96-well plate and the absorbance of the reaction mixture was read at 540 nm using a microplate reader (Synergy-HT Bio-Tek PowerWave XS2, Winooski, Vermont, USA). A blank was prepared for each fucoidan concentration used by preparing the reaction mixture in the same way and replacing α-amylase and starch solution with SPB. This is to avoid any interference caused by the color of fucoidan. A control sample was prepared by replacing α-amylase with an equivalent volume of SPB.

A standard curve of various concentrations of maltose was constructed to quantify the amount of liberated maltose. For each concentration of maltose, 100 μl of maltose solution was allowed to react with 100 μl of DNS color reagent solution. The reaction mixture was heated at 100°C for 15 min and cooled in an ice water bath. Aliquots of 200 μl of reaction mixture were transferred into a 96-well microplate and the absorbance of the reaction mixtures was read at 540 nm using a microplate reader. The standard curve was constructed by plotting a graph of absorbance at 540 nm against concentration of maltose standard. The absorbance of the sample reaction mixtures was then fitted into the standard curve to determine enzyme activity.

### Inhibition Assay for α-Glucosidase Activity

The inhibition activity of fucoidan against α-glucosidase was determined by measuring the amount of *p*-nitrophenol liberated from 4-nitrophenyl α-D- glucopyranoside according to the method by Kazeem et al. (2013) with slight modifications. A series of test on various substrate (4-nitrophenyl α-D- glucopyranoside) and inhibitor (fucoidan) concentrations were conducted to determine the inhibition type. The working enzyme solution was prepared freshly prior to use by diluting the stock α-glucosidase solution (Megazyme, Ireland) to 1 U/ml in sodium phosphate buffer (SPB, 0.1 M, pH 6.9). The substrate solutions of various concentrations (2.5, 5, 10, 15 mM) were prepared by solubilizing 4-nitrophenyl α-D- glucopyranoside into SPB. The fucoidan solutions of various concentrations (0.1, 0.2, 0.5, 1 mg/ml) were prepared by solubilizing the fucoidan samples in SPB.

Aliquots of fucoidan solution of 120 μl, α-glucosidase solution of 60 μl, and SPB of 300 μl were added into a 2 ml Eppendorf tube and incubated at 37°C water bath for 15 min to allow for interactions between the enzyme and fucoidan. The enzyme reaction was initiated by adding 120 μl of substrate solution into the Eppendorf tube containing the enzyme-fucoidan reaction mixture. The reaction was allowed to proceed for 10 min at 37°C. Subsequently, 300 μl of Na_2_CO_3_ solution was added into the Eppendorf tube. Aliquots of 200 μl of reaction mixture were transferred into a 96-well plate and the absorbance of the reaction mixture was read at 405 nm using a microplate reader (Synergy-HT Bio-Tek PowerWave XS2, Winooski, Vermont, USA). A blank was prepared for each fucoidan concentration used by preparing the reaction mixture in the same way and replacing α-glucosidase and 4-nitrophenyl α-D- glucopyranoside solution with SPB. This is to avoid any interference caused by the color of fucoidan. A control sample was prepared by replacing α-glucosidase with an equivalent volume of SPB.

A standard curve of various concentrations of *p*-nitrophenol was constructed. The absorbance of the sample reaction mixtures was fitted into the standard curve to determine enzyme activity.

### Inhibition Assay for Amyloglucosidase Activity

The inhibition activity of fucoidan against amyloglucosidase was determined by measuring the reducing power of released reducing sugar from soluble starch according to the method by Onofre et al. (2012) with slight modification. The working enzyme solution was prepared freshly prior to use by diluting the stock amyloglucosidase solution (Megazyme, Ireland) to 6.5 U/ml in sodium phosphate buffer (SPB, 0.1 M, pH 6.9). The substrate solutions of various concentrations (2.5, 5, 10, 15 mg/ml) were prepared by solubilizing cornstarch into SPB and gelatinized at 100°C for 15 min. The substrate solutions were allowed to cool to room temperature prior to use. The fucoidan solutions of various concentrations (0.5, 1, 1.5, 2 mg/ml) were prepared by solubilizing the fucoidan samples in SPB.

Aliquots of fucoidan solution of 20 μl and amyloglucosidase solution of 20 μl were added into a 2 ml Eppendorf tube and incubated at 37°C water bath for 15 min to allow for interactions between the enzyme and fucoidan. The reaction was initiated by adding 60 μl of starch solution into the Eppendorf tube and the reaction was allowed to proceed for 5 min at 37°C. Subsequently, 100 μl of DNS color reagent was added into the Eppendorf tube and incubated at 100°C for 15 min to allow for color development. The reaction mixture was cooled in an ice water bath and 200 μl of reaction mixture was transferred into a 96-well plate. The absorbance of the reaction mixture was read at 540 nm using a microplate reader (Synergy-HT Bio-Tek PowerWave XS2, Winooski, Vermont, USA). A blank was prepared for each fucoidan concentration used by preparing the reaction mixture in the same way and replacing amyloglucosidase and starch solution with SPB. A control sample was prepared by replacing amyloglucosidase with an equivalent volume of SPB.

A standard curve of various concentrations of maltose was constructed to quantify the amount of liberated maltose. The absorbance of the sample reaction mixtures was fitted into the standard curve to determine enzyme activity.

### Determination of Type of Enzyme Inhibition

The type of enzyme inhibition was determined graphically using the Michaelis-Menten plot and Lineweaver-burk plot. The data sets obtained from each enzyme inhibition assay were analyzed using the nonlinear regression curve fit in GraphPad Prism software (Intuitive Software for Science, San Diego, CA) to determine maximal velocity (*V_Max_*) and the Michaelis-Menten constant (*K_M_*).

### Statistical Analysis

All the analysis was performed in triplicates. The results are expressed as the mean values of the triplicate measurements together with their standard deviations. Statistical analysis was conducted by one-way ANOVA using SPSS 21 software (IBM Corporation, New York, USA). *Post-hoc* analysis using Duncan’s multiple-range test was also conducted to determine if there were significant differences (p < 0.05) between the samples.

## Results and Discussion

### Inhibition Type of Fucoidan Against α-Amylase

**Figure 1:**
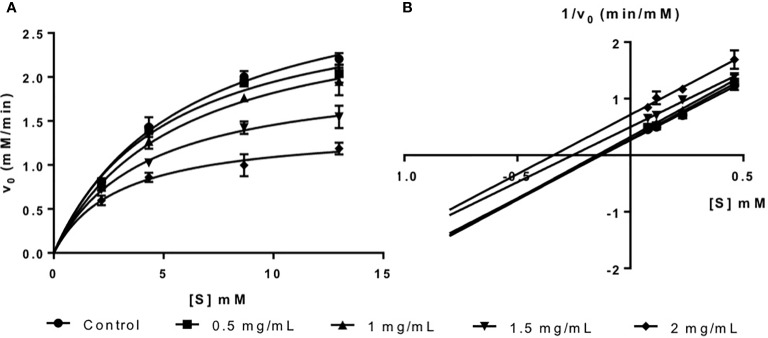
**(A)** Michaelis-Menten plot and **(B)** Lineweaver-Burk plot of fucoidan inhibition against α-amylase.

The inhibition activity of fucoidan against α-amylase was demonstrated in this study *via* the Michaelis-Menten plot in **Figure 1A**. This is in consistent with most literature reports where fucoidans from different seaweed species were found to be inhibitors of α-amylase (Cho et al., 2011; Kim et al., 2014; Lakshmanasenthil et al., 2014).

The inhibition type of fucoidan against α-amylase was determined by the Lineweaver-Burk (double-reciprocal) plot as shown in **Figure 1B**. It was observed that for each concentration of fucoidan, all the lines in the Lineweaver-Burk plot (**Figure 1B**) have similar slopes (i.e. parallel), with different y-intercepts. This suggests that the type of inhibition was uncompetitive. In order to further confirm the type of inhibition of fucoidan against α-amylase, the maximal velocity (*V_Max_*) and Michaelis-Menten constant (*K_M_*) values were obtained and tabulated as shown in **Table 1**. It was observed that both *V_Max_* and *K_M_* decreased significantly with increasing concentration of fucoidan. This is characteristic of an uncompetitive inhibitor (Ramsay and Tipton, 2017). In uncompetitive enzyme inhibition, the inhibitor binds to enzyme at infinitely high substrate concentration (Waldrop, 2009). This implies that an uncompetitive inhibitor does not bind to free enzyme, but instead, binds only to the enzyme-substrate complex (Ramsay and Tipton, 2017). Therefore as the concentration of the uncompetitive inhibitor increases, more of the enzyme is converted to the inactive form (where it is bound to both substrate and inhibitor i.e. E-S-I form) (Ramsay and Tipton, 2017). This will in turn lead to a significant decrease in both *V_Max_* and *K_M_*, by almost the same magnitude, producing parallel lines in the Lineweaver-Burk plot as shown in **Figure 1B** (Ramsay and Tipton, 2017).

**Table 1:** Inhibition activity of fucoidan against α-amylase—Maximal velocity (*V_Max_*) and Michaelis-Menten constant (*K_M_*).

Concentration of fucoidan (mg/ml)	*V_Max_* (mM/min)	*K_M_* (mM)
Control	3.274 ± 0.1007^a^	5.893 ± 0.4187^a^
0.5	2.948 ± 0.1016^ab^	5.158 ± 0.4344^ab^
1.0	2.834 ± 0.1022^b^	5.577 ± 0.475^ab^
1.5	2.058 ± 0.0631^c^	4.136 ± 0.3418^bc^
2.0	1.415 ± 0.05508^d^	2.983 ± 0.3641^c^

^a–d^Values are presented as mean with standard deviation (n = 9). Within each column, mean values with different superscript lowercase letters are statistically different (p < 0.05) across the different samples.

### Inhibition Type of Fucoidan Against α-Glucosidase

**Figure 2:**
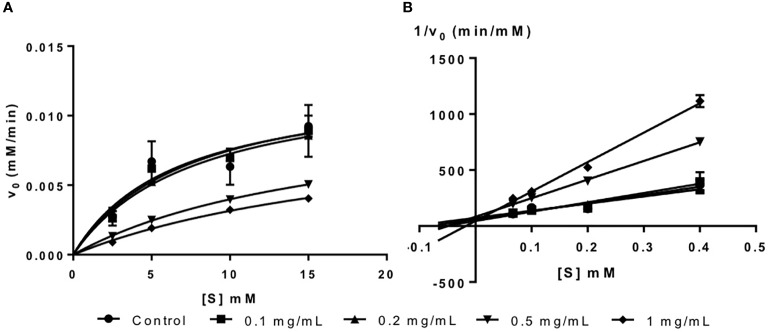
**(A)** Michaelis-Menten plot and **(B)** Lineweaver-Burk plot of fucoidan inhibition against α-glucosidase.

Fucoidan from *U. pinnatifida* is an inhibitor of α-glucosidase as shown in the Michaelis-Menten plot in **Figure 2A**. Likewise, it has been reported in the literature that fucoidan from various seaweed species are inhibitors of α-glucosidase (Kim et al., 2014; Kumar et al., 2015).

The inhibition type of fucoidan against α-glucosidase was determined by the Lineweaver-Burk (double-reciprocal) plot as shown in **Figure 2B**. It was observed that for each concentration of fucoidan, all the lines in the Lineweaver-Burk plot (**Figure 2B**) had almost similar y-intercepts, with different slopes and x-intercepts. This suggests that the type of inhibition was competitive since competitive inhibitor only affects the slope of the Lineweaver-Burk plot but not the y-intercept (Waldrop, 2009). In order to further confirm the type of inhibition of fucoidan against α-glucosidase, the *V_Max_* and *K_M_* values were obtained and tabulated as shown in **Table 2**. It was observed that only *K_M_* decreased significantly with increasing concentration of fucoidan while *V_Max_* did not change significantly with increasing fucoidan concentration. This is characteristic of a competitive inhibitor (Ramsay and Tipton, 2017). In competitive enzyme inhibition, the inhibitor binds to enzyme at its active site, which is the same site where substrate binds to the enzyme (Ramsay and Tipton, 2017). Thus, both substrate and inhibitor share the same binding site on enzyme. However, the enzyme can only bind to either the substrate or inhibitor at any one time. Therefore, at very high substrate concentration, the inhibitor will be displaced from the active site of enzyme. As such, the maximal velocity, which is the reciprocal of the y-intercept of the Lineweaver-Burk plot, is unaffected in the presence of a competitive inhibitor as shown in **Figure 2B** and **Table 2** (Waldrop, 2009; Ramsay and Tipton, 2017).

**Table 2:** Inhibition activity of fucoidan against α-glucosidase—Maximal velocity (*V_Max_*) and Michaelis-Menten constant (*K_M_*).

Concentration of fucoidan (mg/ml)	*V_Max_* (mM/min)	*K_M_* (mM)
Control	0.0126 ± 0.0016^a^	6.594 ± 1.921^a^
0.1	0.0131 ± 0.0014^a^	7.458 ± 1.768^a^
0.2	0.0129 ± 0.0004^a^	7.767 ± 0.507^a^
0.5	0.0108 ± 0.0005^a^	17.16 ± 1.235^ab^
1.0	0.0101 ± 0.0006^a^	21.89 ± 2.007^b^

^a–b^Values are presented as mean with standard deviation (n = 9). Within each column, mean values with different superscript lowercase letters are statistically different (p < 0.05) across the different samples.

### Inhibition Type of Fucoidan Against Amyloglucosidase

**Figure 3:**
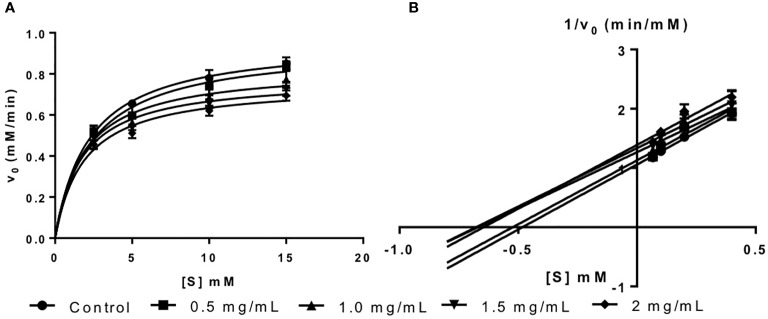
**(A)** Michaelis-Menten plot and **(B)** Lineweaver-Burk plot of fucoidan inhibition against amyloglucosidase.

The inhibition activity of fucoidan against amyloglucosidase was demonstrated in this study *via* the Michaelis-Menten plot in **Figure 3A**. This is in consistent with the results reported by Cho et al. (2011) where fucoidan isolated from *U. pinnatifida* also exhibited inhibitory effects against amyloglucosidase activity.

The inhibition type of fucoidan against amyloglucosidase was determined by the Lineweaver-Burk (double-reciprocal) plot as shown in **Figure 3B**. It was observed that for each concentration of fucoidan, all the lines in the Lineweaver-Burk plot (**Figure 3B**) have similar slopes (i.e. parallel), with different y-intercepts, similar to that of the Lineweaver-Burk plot of α-amylase. This suggests that the type of inhibition was uncompetitive. In order to further confirm the type of inhibition of fucoidan against amyloglucosidase, the maximal velocity (*V_Max_*) and Michaelis-Menten constant (*K_M_*) values were obtained and tabulated as shown in **Table 3**. It was observed that both *V_Max_* and *K_M_* decreased significantly with increasing concentration of fucoidan. This further confirms that the fucoidan was an uncompetitive inhibitor of amyloglucosidase (Ramsay and Tipton, 2017).

**Table 3:** Inhibition activity of fucoidan against amyloglucosidase—Maximal velocity (*V_Max_*) and Michaelis-Menten constant (*K_M_*).

Concentration of fucoidan (mg/ml)	*V_Max_* (mM/min)	*K_M_* (mM)
Control	0.962 ± 0.014^a^	2.21 ± 0.13^a^
0.5	0.938 ± 0.025^a^	2.36 ± 0.24^a^
1.0	0.832 ± 0.024^b^	1.85 ± 0.23^b^
1.5	0.781 ± 0.024^c^	1.73 ± 0.24^b^
2.0	0.753 ± 0.016^c^	1.87 ± 0.17^b^

^a–c^Values are presented as mean with standard deviation (n = 9). Within each column, mean values with different superscript lowercase letters are statistically different (p < 0.05) across the different samples.

### Inhibition of Starch Hydrolases by Fucoidan from *U. pinnatifida*

Fucoidan extracted from U. pinnatifida has been demonstrated in Inhibition Type of Fucoidan Against α-Amylase, Inhibition Type of Fucoidan Against α-Glucosidase, and Inhibition Type of Fucoidan Against Amyloglucosidase to exhibit inhibitory effects against the three key starch hydrolases—α-amylase, α-glucosidase, and amyloglucosidase. However, the exact mechanisms by which fucoidan inhibited the three different starch hydrolases has yet to be fully elucidated. Moreover, it was observed that fucoidan from U. pinnatifida exhibited differential inhibitory potency against the three different starch hydrolases.

Kim et al. (2014) reported that the inhibitions on α-amylase and α-glucosidase were differentially modulated by fucoidan from *F. vesiculosus* and *A. nodosum*. It was reported that fucoidan from *A. nodosum* exhibited inhibition against α-amylase while fucoidan from *F. vesiculosus* did not (Kim et al., 2014). In addition, Kim et al. (2014) also reported that the level of inhibition by fucoidan against α-amylase activity was dependent upon the harvest period of seaweed, which in turn influenced the chemical structure of fucoidan (e.g. ratio of L-fucose to other monosaccharides in the backbone structure, sulfate content, monosaccharide content, and uronic acid content). While fucoidan from both *F. vesiculosus* and *A. nodosum* are largely composed of α-(1–3) linked sulfated L-fucose units in its backbone structure, a low proportion α-(1–4) linked fucose or a repeating α-(1–3) and α-(1–4)-linkage is only found in the backbone structure of fucoidan from *A. nodosum* (Patankar et al., 1993; Daniel et al., 1999; Chevolot et al., 2001; Marais and Joseleau, 2001). Thus it has been suggested that the backbone structure of fucoidan plays an important role in determining its inhibitory activity against α-amylase (Kim et al., 2014). In this study, fucoidan from New Zealand *U. pinnatifida* was composed of a repeating backbone structure of alternatively linked α(1–3) and α(1–4) fucose and galactose units, with a high degree of sulfation (Lu et al., 2018; Koh et al., 2019). Thus it should also exhibit potent inhibitory activity against α-amylase.

Moreover, the inhibition effect of fucoidan against α-amylase has been reported to be dependent on its sulfate content (Cho et al., 2011). Cho et al. (2011) reported that a sulfate content of ≥51% was required for the α-amylase inhibition activity by fucoidan isolated from Korean brown seaweed *U. pinnatifida*, and demonstrated that fucoidan with a sulfate content of 42% did not exhibit inhibitory activity against α-amylase (Cho et al., 2011). However, in our study, the fucoidan from New Zealand *U. pinnatifida* with an average sulfate content of only 22.83 ± 1.00 (w/w) % exhibited significantly inhibitory effects against α-amylase. Thus this suggests that while sulfate content might play a role in α-amylase inhibition, it has to be considered together with other factors or structural features involved in the α-amylase inhibitory activities of sulfated polysaccharides like fucoidan.

In the literature, it was proposed that fucoidan exhibited α-glucosidase inhibition *via* its hydrogen scavenging activity, which is similar to the α-glucosidase inhibition mechanism of most polyphenolic compounds (Kim et al., 2014). The hydrolysis of α(1–4) glucosidic bonds by α-glucosidase requires the presence of hydrogen at the active site of α-glucosidase. Thus, it has been proposed that α-glucosidase inhibitor such as fucoidan scavenges the hydrogen ion at the catalytic site of α-glucosidase thereby inhibiting the enzymatic activity (Borges de Melo et al., 2006; Mohan and Pinto, 2007). Another mechanism by which fucoidan may inhibit α-glucosidase activity is by mimicking the enzyme substrate, similar to the mode of action of acarbose (Rabasa-Lhoret and Chiasson, 2003). It is reported in our study that fucoidan from *U. pinnatifida* is a competitive inhibitor of α-glucosidase that competed with the substrate and bind to the active sites of the enzyme molecule. The binding of fucoidan to the active sites of α-glucosidase might be modulated by electrostatic interactions between the negatively charged sulfate groups of fucoidan and the enzyme. This would account for the significantly higher α-glucosidase inhibitory activities of over-sulfated fucoidan reported in the literature (Cho et al., 2011). Thus this suggests that the conformation and electrostatic charges on the fucoidan molecule may participate in active site binding to inhibit the activity of α-glucosidase (Heightman and Vasella, 1999; Krasikov et al., 2001; Lillelund et al., 2002).

As reported in this study, fucoidan from *U. pinnatifida* was an uncompetitive inhibitor of α-amylase and amyloglucosidase. This implies that fucoidan bound to the enzyme-substrate complex instead of binding to the enzyme itself (either active site or other binding sites). This is similar to the inhibition mechanism of acarbose on α-amylase reported by Oudjeriouat et al. (2003). It was reported that acarbose had a much poorer affinity for the active sites of α-amylase as compared to the affinity of substrates to the α-amylase active sites. As such, in the presence of both substrate and inhibitor, the enzyme-substrate complex would be formed at much higher rate than that of the enzyme-inhibitor complex (Oudjeriouat et al., 2003). The formation of the enzyme-substrate complex in turn activated a secondary binding site on α-amylase, for which the inhibitor acarbose had high affinity to, thereby forming the enzyme-substrate-inhibitor complex which inhibited the activity of the enzyme (Oudjeriouat et al., 2003). Therefore, it can be proposed that fucoidan also binds to the secondary binding site on the enzyme that is only functional when substrate binds to its active site, and thus inhibits the activity of α-amylase.

A similar mechanism can be proposed for the uncompetitive inhibition of amyloglucosidase by fucoidan from *U. pinnatifida*. Moreover, in the case of amyloglucosidase, the binding of fucoidan to the secondary binding site of enzyme could be facilitated by the electrostatic interactions between the negatively charged sulfate groups of fucoidan and the enzyme’s secondary binding site. This may also account for the enhanced amyloglucosidase inhibitory effects of fucoidan with higher sulfate content reported in the literature (Cho et al., 2011). Another possible mechanism by which fucoidan inhibits amyloglucosidase activity is by slowing down the diffusion of glucose from the active site of enzyme as a result of the viscosity of fucoidan (Ou et al., 2001; Maki et al., 2007; Rioux et al., 2007). Overall, fucoidan may inhibit α-amylase and amyloglucosidase through binding at a secondary site of the enzyme-substrate complex *via* electrostatic interactions involving sulfate groups of fucoidan, as well as by increasing the viscosity of the reaction medium.

### Potential of Fucoidan as a T2DM Control and Management Agent

The inhibitory effects of fucoidan against α-amylase, α-glucosidase, and amyloglucosidase highlight the potential of fucoidan to be used as an anti-diabetic agent. Currently, there are many commercially available drugs in the market for diabetes mellitus treatment such as acarbose. However, antidiabetic drugs e.g. acarbose have numerous side effects such as abdominal distension, flatulence, meteorism, and diarrhea as a result of the strong inhibitory effects of acarbose against α-amylase (Puls and Keup, 1975; Bishoff, 1985). Acarbose strongly inhibits the activity of pancreatic α-amylase, resulting in the buildup of undigested carbohydrates in the colon (Bishoff, 1985; Horii et al., 1986). Consequently, excessive and abnormal bacterial fermentation of the undigested carbohydrates occurs in the colon, leading to the above-mentioned side effects of acarbose (Bishoff, 1985; Horii et al., 1986).

One way to reduce the adverse effects associated with acarbose is to replace it with an inhibitor that is more potent against α-glucosidase than α-amylase (Cho et al., 2011). Thus, many researches are focused on characterizing natural extracts with strong α-glucosidase inhibition activity as potential anti-diabetic agents and for diabetes prevention. While excessive α-amylase inhibition may trigger many adverse effects, partial inhibition of α-amylase is still beneficial in modulating the rate of glucose release from starch.

In this study, it was observed that the IC_50_ of fucoidan inhibition against α-glucosidase was significantly lower than those against α-amylase and amyloglucosidase, despite of a higher substrate concentration (**Table 4**). While the substrate used for both assays were different, the higher IC_50_ value for fucoidan inhibition of glucosidase suggests that *U. pinnatifida* is likely to have more potent inhibitory effects against α-glucosidase than α-amylase. This further expounds the benefits of fucoidan from *U. pinnatida* in diabetes prevention and management as compared to the popular anti-diabetic drugs such as acarbose. However, further works need to be conducted to validate the different potency of *U. pinnatifida* fucoidan on α-amylase and α-glucosidase.

**Table 4:** Type of inhibition of fucoidan against α-amylase, α-glucosidase, and amyloglucosidase.

		[Substrate] mM	IC_50_ (mg/ml)	Type of inhibition
α-Amylase		4.33	0.190 ± 0.005^b^	Uncompetitive
α-Glucosidase		10.00	0.137 ± 0.012^a^	Competitive
Amyloglucosidase		4.33	0.280 ± 0.016^c^	Uncompetitive

^a–c^Values are presented as mean with standard deviation (n = 9). Within each column, mean values with different superscript lowercase letters are statistically different (p < 0.05) across the different samples.

## Conclusions

This study demonstrated the inhibitory effects of fucoidan isolated from New Zealand *U. pinnatifida* against three key starch hydrolases—α-amylase, α-glucosidase, and amyloglucosidase. Based on the Lineweaver-Burk plots, as well as maximal velocity and Michaelis-Menten constant, it is concluded that fucoidan from *U. pinnatifida* is an uncompetitive inhibitor of both α-amylase and amyloglucosidase, and a competitive inhibitor of α-glucosidase. In addition, based on the IC_50_ values, it has been demonstrated that fucoidan from *U. pinnatifida* is likely to exhibit significantly stronger inhibitory effects against α-glucosidase than α-amylase and amyloglucosidase. As such, fucoidan from *U. pinnatifida* possesses the desirable attributes as a potential anti-diabetic agent and offers new ideas in diabetes management and prevention.

## Data Availability Statement

All datasets generated for this study are included in the article/supplementary material.

## Author Contributions

JL and WZ conceived project. HK carried out the experiment. JL provided materials. WZ provided resources. JL and WZ supervised HK. HK analyzed the data and wrote the manuscript. JL and WZ edited the manuscript. All authors have read and agreed to submit this manuscript for publication.

## Conflict of Interest

The authors declare that the research was conducted in the absence of any commercial or financial relationships that could be construed as a potential conflict of interest.
